# Chloride Transport Resistance, Concrete Cover Performance, and Service-Life Assessment of Reinforced Concrete under Brazilian Durability Requirements

**DOI:** 10.1021/acsomega.6c08228

**Published:** 2026-10-20

**Authors:** Thalles Murilo Santos de Almeida, Wanderson Santos de Jesus, Flávia Araujo Azevedo, Marcelo Tramontin Souza, José Renato de Castro Pessôa

**Affiliations:** Department of Engineering and Computing, State University of Santa Cruz (UESC), 45662-900 Ilhéus, BA, Brazil

## Abstract

Chloride-induced corrosion is one of the main causes of premature deterioration in reinforced concrete structures exposed to marine and industrial environments. In such conditions, durability depends not only on the quality and transport resistance of the cementitious matrix but also on the adequacy of the reinforcement cover thickness. Although ABNT NBR 6118:2026 prescribes minimum concrete cover requirements according to environmental exposure classes, experimental validations of these recommendations under severe chloride exposure conditions remain limited. This study evaluates the effectiveness of the minimum reinforcement cover thicknesses specified for high environmental aggressiveness classes (EC III and EC IV) through accelerated chloride migration testing and the analysis of concrete quality parameters. Concrete specimens were produced using ordinary Portland cement (PC V ARI RS) with water-to-cement (w/c) ratios ranging from 0.43 to 0.68. The experimental program included compressive strength, ultrasonic pulse velocity (UPV), water absorption, void index, chloride penetration depth, and non-steady-state chloride migration coefficient (*D*_ns_) measurements. Chloride penetration fronts were determined by colorimetric analysis after accelerated migration testing and compared with the nominal concrete cover thicknesses prescribed by ABNT NBR 6118:2026. The results demonstrated that all investigated mixtures presented chloride penetration depths lower than the minimum cover thicknesses specified for EC III and EC IV, even for concretes that exceeded the maximum w/c ratio limits established by the standard, indicating the conservative nature of the Brazilian prescriptive durability approach. Chloride penetration depths decreased from approximately 25 mm to 13 mm as the w/c ratio decreased, while *D*_ns_ values were reduced from 12.08 × 10^–12^ to 4.11 × 10^–12^ m^2^/s, indicating substantial improvements in chloride transport resistance due to matrix densification and reduced transport pathways. Strong correlations were observed between chloride penetration behavior and *D*_ns_, compressive strength, void index, and UPV, demonstrating the influence of pore connectivity and internal concrete quality on chloride transport. Additionally, the experimentally obtained *D*_ns_ values allowed comparative interpretations regarding the long-term chloride resistance and potential service-life performance of the investigated concretes.

## Graphical abstract

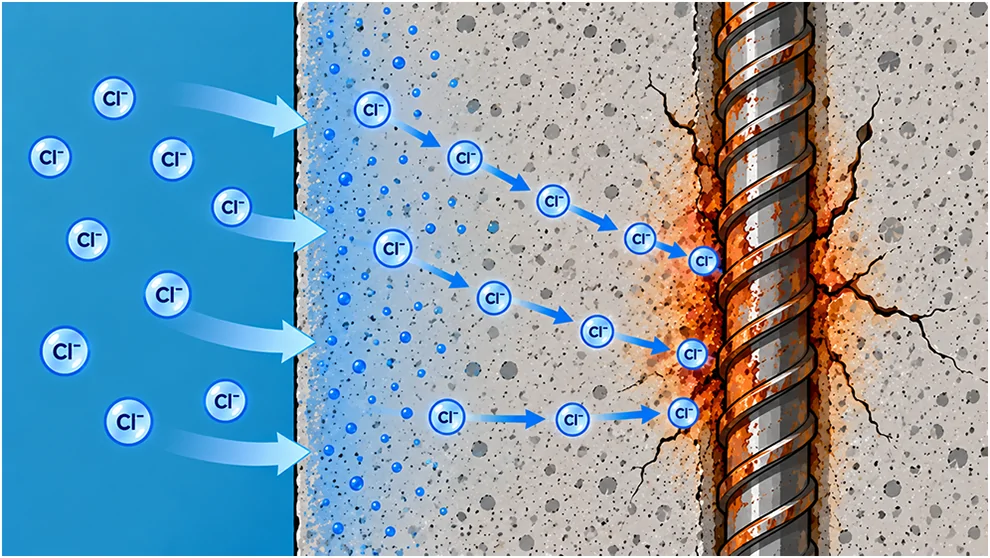

## 1 Introduction

The durability of reinforced concrete structures exposed to aggressive environments is strongly affected by the ingress of chloride ions, which can initiate reinforcement depassivation and corrosion. Chloride-induced deterioration is particularly relevant in marine and industrial environments, where it may lead to cracking, concrete cover spalling, loss of reinforcement cross section, and increased maintenance requirements.^1,2^ Consequently, controlling chloride ingress is a fundamental aspect of the durability-oriented concrete design.

Chloride transport through concrete is primarily governed by the characteristics of its pore structure, including porosity, connectivity, and tortuosity. These characteristics are strongly influenced by the water-to-cement ratio, the curing conditions, and the binder composition. In general, reducing the water-to-cement ratio and producing a denser cementitious matrix decrease ionic transport and improve resistance to chloride ingress.^3–6^ In addition to concrete quality, the thickness of the concrete cover provides a physical barrier that delays the arrival of chloride ions at the reinforcement.

In Brazil, durability requirements for reinforced concrete are established by ABNT NBR 6118,^7^ which adopts a prescriptive approach based mainly on environmental exposure class, minimum compressive strength, maximum water-to-cement ratio, minimum cement content, and nominal reinforcement cover. For highly aggressive environments classified as EC III and EC IV, increasingly restrictive requirements are specified to reduce the risk of chloride-induced deterioration. However, the relationship between the prescribed cover thicknesses and the actual chloride transport resistance of concretes with different quality levels remains insufficiently investigated, particularly under accelerated conditions that are representative of severe chloride exposure.

Accelerated chloride migration tests provide a practical means of evaluating chloride transport over a relatively short experimental period. The non-steady-state chloride migration coefficient (*D*_ns_), commonly determined using procedures derived from NT Build 492,^8^ provides a quantitative parameter for comparing the chloride transport resistance of different concrete mixtures. The chloride penetration depth obtained after accelerated migration can also be directly compared with the reinforcement cover prescribed by durability standards. In addition, properties such as compressive strength, water absorption, void index, and ultrasonic pulse velocity can provide complementary information about matrix densification and internal concrete quality.^9–11^

Despite the extensive use of prescriptive durability requirements, there is still a need for experimental studies that directly relate concrete cover requirements to measured chloride penetration and transport resistance.^12,13^ In particular, the influence of concrete quality over a broad range of water-to-cement ratios and the relationship among chloride penetration, *D*_ns_, and conventional concrete quality indicators require further evaluation. Addressing these aspects can contribute to a better understanding of the relationship between prescriptive durability requirements and the actual chloride transport behavior of the concrete.

Service-life models based on Fick’s second law provide a useful framework for estimating the time required for chloride concentrations at the reinforcement depth to reach a critical threshold. In principle, service-life assessments based on natural chloride diffusion coefficients obtained under long-term exposure conditions can provide a more representative characterization of chloride transport under field-relevant conditions. However, determining natural diffusion coefficients experimentally requires prolonged exposure periods, often involving months or years of testing, which limits their practical applicability for comparative experimental studies. Accelerated chloride migration tests, in contrast, allow transport resistance to be evaluated within substantially shorter testing periods and therefore provide a practical alternative for obtaining comparative transport parameters. Nevertheless, the application of accelerated migration results to natural diffusion and service-life prediction introduces uncertainties because the applied electrical field accelerates ionic transport and does not fully reproduce the mechanisms governing chloride ingress under natural exposure. Consequently, service-life estimates based on accelerated test results should be interpreted with caution, particularly when extrapolating laboratory measurements to long-term field conditions.

In the present study, service-life estimates are therefore used primarily as comparative indicators of the potential long-term durability of the investigated concretes. The calculations are based on experimentally determined *D*_ns_ values and literature-based assumptions for parameters, such as the aging factor, surface chloride concentration, critical chloride threshold, and exposure conditions. These assumptions are necessary to enable comparative modeling but do not fully reproduce the variability of the natural exposure.

Therefore, this study evaluates the effectiveness of the minimum reinforcement cover thicknesses prescribed by ABNT NBR 6118 for environmental exposure classes III and IV by using accelerated chloride migration testing. Concrete mixtures with different water-to-cement ratios were investigated to assess the influence of the matrix quality on chloride penetration and transport resistance. The relationships among chloride penetration depth, *D*_ns_, compressive strength, void index, and UPV were also evaluated. Finally, Fick-based service-life estimates were performed using the experimentally obtained transport parameters and explicitly stated literature-based assumptions, with the purpose of providing a comparative assessment of the potential long-term durability of the investigated concretes under different chloride-rich exposure conditions.

## 2 Comparison of Durability Requirements and Prescriptive Constraints between Brazilian, European, and American Standards for Chloride-Exposed Reinforced Concrete Structures

The durability of reinforced concrete structures exposed to chloride-rich environments is strongly dependent on the combined effects of the concrete quality, transport resistance, and reinforcement protection. In Brazil, these requirements are established by ABNT NBR 6118:2026,^7^ which adopts a prescriptive durability-based approach considering environmental exposure conditions, minimum compressive strength, maximum water-to-cement ratio, minimum cement content, and nominal concrete cover thicknesses.

The Brazilian standard classifies reinforced concrete structures according to environmental exposure classes (ECs), which represent the aggressiveness of the surrounding environment and the potential risk of deterioration mechanisms such as carbonation, chloride ingress, and reinforcement corrosion. Exposure classes III and IV correspond to highly aggressive and very highly aggressive environments, respectively, including marine regions, industrial atmospheres, splash zones, and areas subjected to high chloride contamination.

To mitigate chloride-induced deterioration, ABNT NBR 6118:2026 prescribes increasingly restrictive durability requirements as the environmental aggressiveness increases. For reinforced concrete structures exposed to EC III and EC IV conditions, minimum compressive strengths of 30 and 40 MPa are recommended, respectively. Simultaneously, the standard limits the maximum allowable water-to-cement ratio and increases the minimum cement content in order to reduce the concrete permeability and improve matrix densification. Additionally, larger nominal concrete cover thicknesses are required to delay chloride penetration and reinforcement depassivation during the design service life of the structure.

For slabs, the prescribed nominal concrete cover thicknesses are 35 mm and 45 mm for exposure classes III and IV, respectively. For beams, columns, and elements in contact with soil, the required cover thicknesses increase to 40 mm and 50 mm for exposure classes III and IV, respectively. These values are intended to provide an adequate physical barrier against aggressive agents and to increase the time required for chloride ions to reach the reinforcing steel. Table 1 summarizes the main durability-related constraints prescribed by ABNT NBR 6118:2026 for each environmental exposure class, including minimum compressive strength, maximum water-to-cement ratio, minimum cement content, and nominal concrete cover thicknesses for different structural elements.

**Table 1:** Durability-Related Requirements Prescribed by ABNT NBR 6118:2023 for Reinforced Concrete Structures according to Aggressive Environmental Exposure Classes (EC), Including Minimum Compressive Strength, Maximum Water-to-Cement Ratio, Minimum Cement Content, and Nominal Concrete Cover Thicknesses for Different Structural Elements

						Nominal concrete cover		
	Environmental exposure class	Typical exposure condition	Minimum compressive strength fck,min (MPa)	Maximum w/c ratio	Minimum cement content (kg/m^3^)	Slabs (mm)	Beams and columns (mm)	Elements in contact with soil (mm)
Standard ABNT NBR 6118:2023	EC III	High aggressiveness–marine and industrial environments	C30	0.55	320	35	40	40
	EC IV	Very high aggressiveness–splash zones, tidal regions, and highly chloride-contaminated environments	C40	0.45	360	45	50	50

The durability philosophy adopted by ABNT NBR 6118:2026 is consistent with the general approach employed by major international standards, such as EN 206/Eurocode 2 ^14^ and ACI 318,^15^ which also establish progressively stricter durability requirements as chloride exposure severity increases. Nevertheless, important differences exist regarding the classification systems, prescriptive parameters, and methodologies adopted to define concrete quality and reinforcement protection requirements. Table 2 presents a comparison between the main durability-related requirements prescribed by European and American standards for reinforced concrete structures exposed to chloride-rich marine environments.

**Table 2:** Durability-Related Requirements Prescribed by European and American Standards for Reinforced Concrete Structures Exposed to Chloride-Rich Marine Environments

Standard	Exposure class	Typical exposure condition	Minimum compressive strength (MPa)	Maximum w/c ratio	Minimum cement content (kg/m^3^)	Typical nominal concrete cover (mm)
EN 206:2013 + Eurocode 2	XS1	Structures exposed to airborne marine chlorides, but not directly in contact with seawater	C30	0.50	300	35
	XS2	Permanently submerged marine structures	C35	0.45	320	40
	XS3	Tidal zones, splash zones, and seawater spray exposure	C40	0.45	360	45–50
ACI 318–19/ACI 201.2R	C1	Moderate chloride exposure–moist environments exposed to chlorides	*f*′c = 35	0.50	Not explicitly specified	40
	C2	Severe chloride exposure–marine structures, splash zones, seawater exposure, and deicing salts	*f*′ = 40	0.40	Not explicitly specified	50–65

In the European framework, EN 206/Eurocode 2 classifies chloride-rich marine environments using exposure classes XS1, XS2, and XS3, corresponding respectively to airborne marine chlorides, permanently submerged structures, and tidal or splash zones exposed to seawater spray. Similar to the Brazilian standard, the European approach prescribes progressively lower maximum water-to-cement ratios, higher compressive strength classes, and increased minimum cement contents as the environmental aggressiveness increases. For instance, the XS3 exposure class, associated with tidal and splash zones, typically requires concrete with strength class C40/50, a maximum water-to-cement ratio of 0.45, and minimum cement contents close to 360 kg/m^3^, values that are comparable to those prescribed by ABNT NBR 6118:2026 for EC IV conditions.

However, unlike the Brazilian standard, Eurocode 2 does not explicitly prescribe nominal concrete cover thicknesses according to structural element categories, such as slabs, beams, and columns, in a simplified tabulated format. Instead, the required cover thickness depends on additional parameters, including structural class, intended design working life, execution tolerance, and reliability level. Consequently, the European methodology may be considered less directly prescriptive and more closely associated with performance-based durability and service-life concepts. Similarly, the American approach adopted by ACI 318 establishes durability requirements according to chloride exposure severity, which is classified mainly as exposure categories C1 and C2. Structures exposed to severe chloride environments, including marine structures, splash zones, and seawater exposure, are generally required to satisfy maximum water-to-cement ratios between 0.40 and 0.50 and minimum specified compressive strengths of approximately 35–40 MPa. Nevertheless, different from both ABNT NBR 6118 and EN 206, ACI 318 does not explicitly prescribe minimum cement contents for chloride exposure classes. Additionally, the minimum concrete cover requirements in ACI 318 are primarily dependent on the reinforcement diameter, member geometry, cast condition, and direct exposure severity, resulting in broader ranges of recommended cover thicknesses instead of fixed values associated with specific structural elements.

Despite these methodological differences, the Brazilian, European, and American standards are fundamentally based on the same durability principle: reducing chloride transport through matrix densification while simultaneously increasing the physical distance between aggressive agents and reinforcing steel. In this context, parameters such as the water-to-cement ratio, compressive strength, cement content, and concrete cover thickness are indirectly used to control permeability, chloride diffusivity, and the long-term risk of reinforcement corrosion. Although these prescriptive durability approaches are widely adopted in engineering practice, experimental validations correlating normative cover thicknesses with actual chloride penetration behavior remain relatively limited, particularly under Brazilian environmental conditions characterized by extensive coastal regions, elevated humidity, and severe marine exposure. Consequently, evaluating the effectiveness of the durability requirements prescribed by ABNT NBR 6118:2026 through accelerated chloride migration testing may contribute to a better understanding of the relationships among concrete quality, chloride transport resistance, and reinforcement protection under aggressive chloride exposure conditions.

In this regard, chloride migration tests constitute an important tool for assessing the effectiveness of the durability requirements prescribed by structural design standards. Parameters such as chloride penetration depth and non-steady-state chloride migration coefficient (*D*_ns_) provide a more direct evaluation of ionic transport resistance and concrete permeability than conventional mechanical parameters alone. Furthermore, these transport-related indicators contribute to a better understanding of the influence of pore structure refinement and matrix densification on chloride ingress and long-term reinforcement protection in aggressive, chloride-rich environments.

## 3 Materials and Methods

### 3.1 Materials and Features

All concrete mixtures were produced using a CP V ARI RS high early strength sulfate-resistant Portland cement. Natural river sand and crushed granite were used as fine and coarse aggregates, respectively, while a polycarboxylate ether (PCE)-based superplasticizer (MC-PowerFlow 1180) was incorporated to improve the workability and consistency of the mixtures, particularly for concretes with reduced water-to-cement (w/c) ratios. Potable water was used for the concrete mixing and curing procedures. The physical properties and technical characteristics of the constituent materials were previously determined according to the corresponding Brazilian standards and manufacturer technical datasheets, as summarized in Table 3.

**Table 3:** Physical Properties and Technical Characteristics of the Materials Used for Concrete Production, Including Cement, Aggregates, Superplasticizer, and Mixing Water

Material	Property/feature	Value/information	Standard/Source
Cement	Type	PC V ARI RS–High early strength sulfate-resistant Portland cement	NBR 16697 ^16^
	Specific gravity	3070 kg/m^3^	Manufacturer technical datasheet
Fine aggregate (natural sand)	Specific gravity	1.85 g/cm^3^	NBR NM 52 ^17^
	Bulk density	1.63 g/cm^3^	NBR 16972 ^18^
	Void index	11.85%	
	Water absorption	17.65%	NBR NM 30 ^19^
	Maximum particle size	1.18 mm	NBR NM 248 ^20^
	Fineness modulus	1.17	
Coarse aggregate (crushed granite)	Specific gravity	2.75 g/cm^3^	NBR NM 53 ^21^
	Bulk density	1.47 g/cm^3^	NBR 16972 ^18^
	Void index	46.52%	
	Water absorption	0.51%	NBR NM 53 ^21^
	Maximum particle size	12.50 mm	NBR NM 248 ^20^
	Fineness modulus	6.27	
Superplasticizer (MC-PowerFlow 1180)	Type	Polycarboxylate ether (PCE)-based superplasticizer	Manufacturer technical datasheet
	Classification	Water-reducing admixture type 2 (RA2)	NBR 11768–3:2019 ^22^
	Density	1.09 kg/L	Manufacturer technical datasheet
	Recommended dosage	0.2–5.0% by cement mass	

The characterization of the aggregates included the determination of the specific gravity, bulk density, void index, water absorption, maximum particle size, and fineness modulus. These properties were used for concrete mixture design and dosage procedures, which are detailed in the next section.

### 3.2 Mix Design

The concrete mixtures were proportioned using the ABCP mix design method, considering an initial target compressive strength of 20 MPa and an initial water-to-cement ratio (w/c) of 0.68. After defining the reference mixture, five additional mixtures were produced by progressively reducing the w/c ratio in increments of 0.05 while maintaining a constant cement and aggregate content. This procedure was adopted to evaluate the influence of matrix densification and concrete quality on the chloride migration behavior and penetration resistance.

The incorporation of the polycarboxylate ether (PCE)-based superplasticizer allowed the production of mixtures with reduced w/c ratios while maintaining an adequate workability and consistency during mixing, molding, and compaction procedures. No target slump value was defined for the mixtures. Instead, the admixture dosage was empirically adjusted according to the minimum workability requirements necessary to ensure the proper homogenization and casting conditions of the cylindrical specimens.

The concrete mixtures were identified according to their respective w/c ratios. Table 4 presents the mix proportions adopted for all mixtures produced in the experimental program.

**Table 4:** Mix Proportions of the Concrete Mixtures Expressed as Mass Ratios Relative to Cement Content^a^

Mixture	Cement	Fine aggregate	Coarse aggregate	w/c ratio	Superplasticizer
M1	1.00	1.7	3.56	0.68	0.000
M2	1.00	1.7	3.56	0.63	0.000
M3	1.00	1.7	3.56	0.58	0.000
M4	1.00	1.7	3.56	0.53	0.002
M5	1.00	1.7	3.56	0.48	0.002
M6	1.00	1.7	3.56	0.43	0.002

a All mixtures used a cement consumption of 310.18 kg/m^3^ of cement.

### 3.3 Specimen Preparation and Curing

The concrete mixtures were prepared in a 150 L capacity concrete mixer following the procedures recommended by NBR 5738.^23^ Cylindrical specimens with dimensions of 100 mm × 200 mm were molded for all experimental tests. The fresh concrete was cast into cylindrical molds in layers and compacted to minimize entrapped air and ensure adequate homogenization of the specimens. After casting, the specimens were kept under laboratory conditions for 24 h. Subsequently, the samples were demolded and subjected to moist curing in a saturated calcium hydroxide solution for 28 days in accordance with NBR 5738.

For each concrete mixture, five specimens were used for each experimental test, including accelerated chloride migration and penetration depth evaluation, compressive strength, ultrasonic pulse velocity (UPV), water absorption, void index, and specific mass. UPV measurements were performed on the same specimens prior to the compressive strength testing.

### 3.4 Accelerated Chloride Migration and Penetration Depth Evaluation

The resistance of the concrete mixtures to chloride ingress was evaluated through an accelerated chloride migration test based on the methodology proposed by Ribeiro et al.^24^ and successfully employed in previous research conducted by de Jesus et al.,^25^ adapted from NT Build 492.^8^ The test was performed after 28 days of curing using cylindrical concrete specimens that were previously saturated under vacuum conditions.

Initially, the cylindrical specimens were sectioned into slices with an approximately 50 mm thickness. The samples were then vacuum saturated to ensure adequate pore filling prior to chloride migration testing. After saturation, the specimens were positioned between the two compartments of the migration cell. One side of the cell contained a sodium chloride (NaCl) solution, whereas the opposite side contained an alkaline solution. An external electrical potential was applied between the compartments to accelerate the transport of chloride ions through the concrete matrix. Figure 1 illustrates the experimental setup adopted for the accelerated chloride migration test.

**Figure 1:**
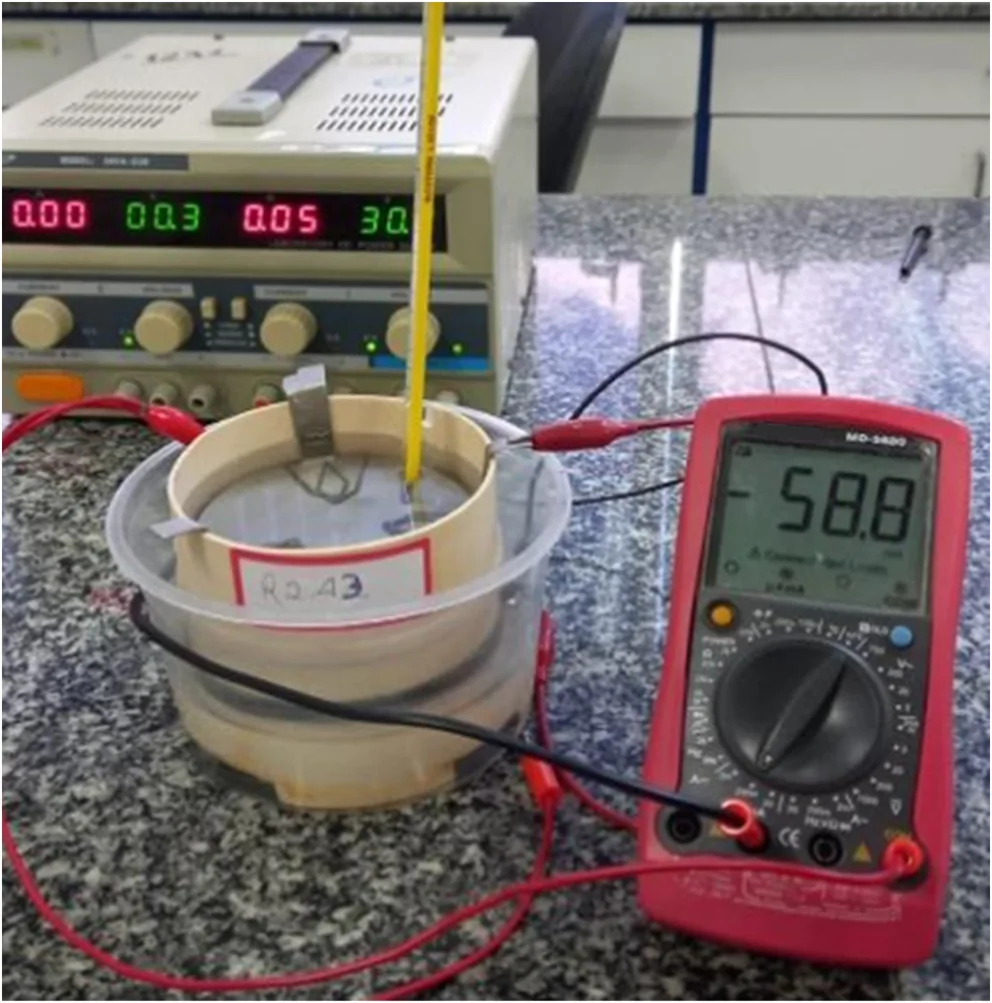
Experimental setup used for the accelerated chloride migration test.

The applied voltage and test duration were defined according to the initial electrical current measured during the test, following the recommendations of the adapted NT Build 492 procedure. Table 5 presents the relationship between the applied voltage and the corresponding test duration adopted in the experimental program.

**Table 5:** Relationship between Applied Voltage and Test Duration Adopted in the Accelerated Chloride Migration Test

Initial current (mA)	Applied voltage (V)	Test duration (h)
*I* < 5	60	96
5 ≤ *I* < 10	60	48
10 ≤ *I* < 30	60	24
30 ≤ *I* < 60	35	24
60 ≤ *I* < 120	20	24
120 ≤ *I* < 180	10	24
180 ≤ *I* < 360	10	6

After completion of the migration test, the specimens were axially split into two halves, and a 0.1 M silver nitrate (AgNO_3_) solution was sprayed onto the freshly fractured surface to reveal the chloride penetration front through a colorimetric indication. The chloride-contaminated region presented a lighter coloration, allowing the visualization of the penetration profile, as illustrated in Figure 2.

**Figure 2:**
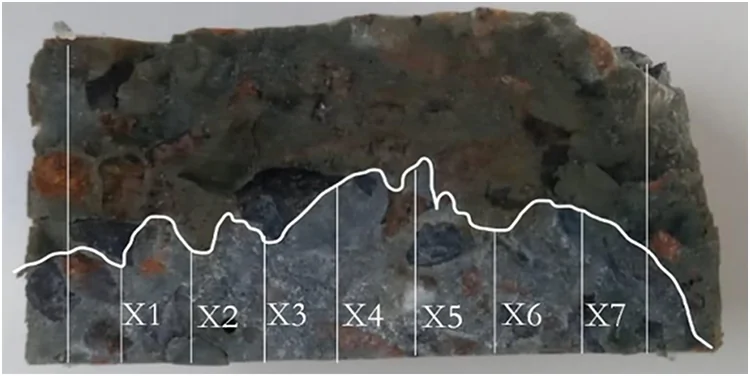
Representative chloride penetration front revealed by AgNO_3_ spraying after the accelerated migration test.

The chloride penetration depth was measured at multiple points along the exposed section of each specimen, allowing the determination of average and maximum penetration depths. Subsequently, the chloride penetration profiles obtained under accelerated conditions were compared with the minimum reinforcement cover thicknesses prescribed by NBR 6118:2026 for environmental aggressiveness classes III and IV. The average chloride penetration depth (*C*_d_) values were also determined. The non-steady-state chloride migration coefficient (*D*_ns_) was determined according to eq 1, adapted from NT Build 492, where *D*_ns_ is the non-steady-state chloride migration coefficient (m^2^ s^–1^), *U* is the applied voltage (V), *T* is the average temperature of the anolyte solution (°C), *L* is the specimen thickness (mm), *X*_d_ is the average chloride penetration depth (mm), and *t* is the test duration (h).

1 $$D_{ns}=\frac{0.0239\left(273+T\right)\times L}{\left(U-2\right)\times t}\left(x_{d}-0.0238\sqrt{\frac{\left(273+T\right)\times L\times X_{d}}{U-2}}\right)$$

### 3.5 Compressive Strength Test

The compressive strength of the concrete mixtures was determined after 28 days of curing in a saturated lime solution, in accordance with NBR 5739, using cylindrical specimens with dimensions of 100 mm × 200 mm. Prior to testing, the specimens were rectified using a diamond saw in order to ensure parallel and regular loading surfaces. The tests were carried out using an EMIC hydraulic compression testing machine, model PC 200C, with a maximum load capacity of 2000 kN.

The axial load was continuously applied at a controlled loading rate of 0.45 ± 0.15 MPa/s, according to the recommendations of NBR 5739,^26^ until specimen failure. The maximum load recorded during the test was used to determine the compressive strength of each specimen.

For each concrete mixture, five specimens were tested, and the reported results correspond to the average compressive strength values obtained for each mixture. The compressive strength results were subsequently correlated with ultrasonic pulse velocity (UPV), void index, and non-steady-state chloride migration coefficient (*D*_ns_) in order to evaluate the influence of concrete quality and matrix densification on chloride transport behavior and durability performance.

### 3.6 Ultrasonic Pulse Velocity

The ultrasonic pulse velocity (UPV) test was performed in accordance with NBR 8802 ^27^ after 28 days of curing in order to evaluate the internal integrity and quality of the concrete mixtures. The measurements were conducted by using an ultrasonic pulse velocity device equipped with transducers operating at frequencies of 54 and 150 kHz.

Prior to testing, the surfaces of the cylindrical specimens (100 mm × 200 mm) were cleaned and prepared to ensure adequate acoustic coupling between the transducers and the concrete surface. A coupling material was applied to minimize signal loss during wave propagation. The measurements were performed using the direct transmission method, in which the transmitting and receiving transducers were positioned on opposite faces of the specimen.

The ultrasonic pulse velocity (UPV, m/s) was calculated by dividing the travel distance by the pulse propagation time recorded by the equipment, as expressed in eq 2, where *L* is the distance traveled by the ultrasonic wave (m) and *t* is the measured pulse travel time (s). For each concrete mixture, five specimens were evaluated, and the reported values correspond to the average UPV results obtained for each transducer frequency.

2 $$\mathrm{UPV}=\frac{L}{t}$$

### 3.7 Water Absorption, Void Index, and Specific Mass

The water absorption, void index, and bulk density of the concrete mixtures were determined after 28 days of curing in accordance with NBR 9778.^28^ Cylindrical specimens with dimensions of 100 mm × 200 mm were used for the tests.

Initially, the specimens were dried in an oven at 105 ± 5 °C until constant mass was achieved. After cooling to room temperature, the dry mass of each specimen was measured. Subsequently, the samples were immersed in water for saturation and later subjected to boiling in water according to the procedure established by the standard. After the saturation process, the saturated mass and submerged mass of the specimens were determined.

The experimental procedure adopted for the determination of water absorption, void index, and density is illustrated in Figure 3. The water absorption by immersion (WA, %), void index (VI, %), and bulk density (ρ, g/cm^3^) were calculated according to eqs 3, 4, and 5, respectively, where *m*_d_ is the dry mass of the specimen (g), *m*_sat_ is the saturated mass (g), and *m*_i_ is the immersed mass (g). For each concrete mixture, five specimens were tested, and the reported values correspond to the average results obtained for each property.

3 $$\mathrm{WA}=\frac{m_{\mathrm{sat}}-m_{d}}{m_{d}}$$

4 $$\mathrm{IV}=\frac{m_{\mathrm{sat}}-m_{d}}{{m_{\mathrm{sat}}-m}_{i}}$$

5 $$\rho =\frac{m_{d}}{{m_{\mathrm{sat}}-m}_{i}}$$

**Figure 3:**
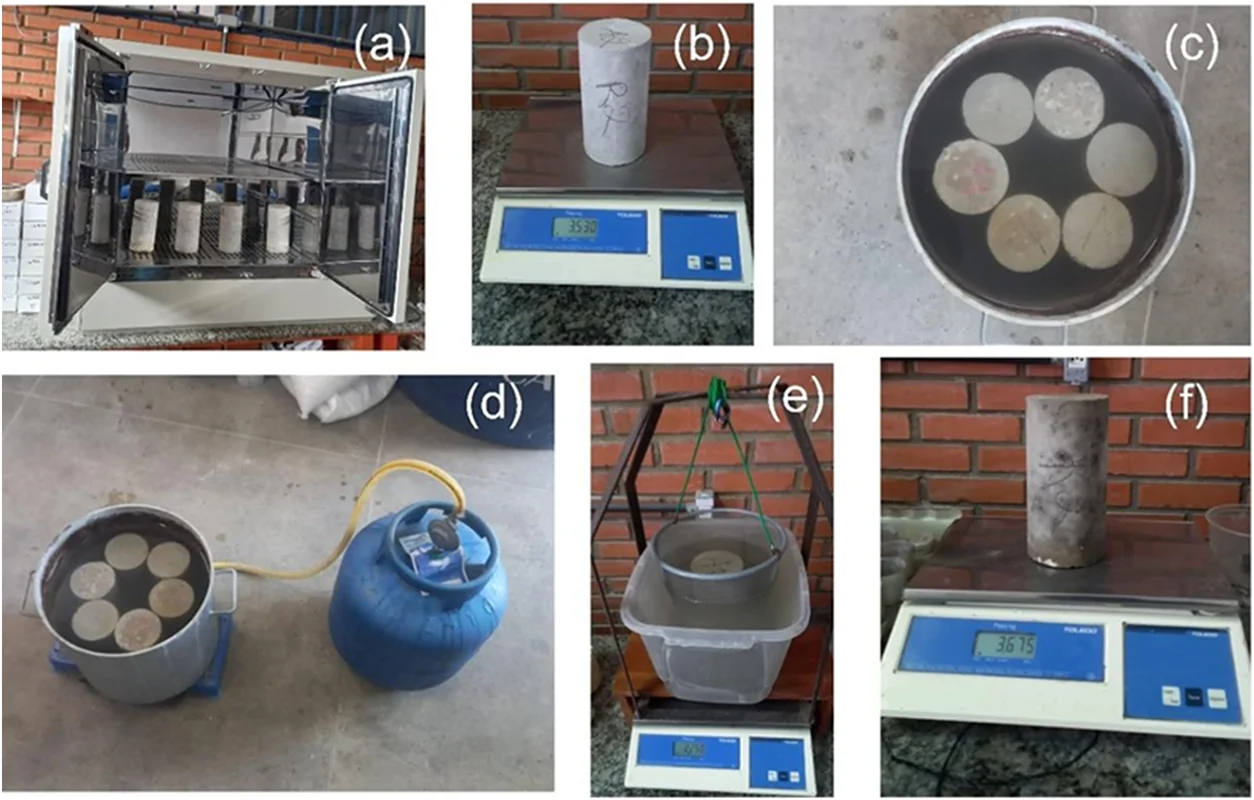
Experimental procedure adopted for the determination of water absorption, void index, and bulk density: (a) oven drying; (b) dry mass measurement; (c) specimen saturation; (d) boiling procedure; (e) immersed mass measurement; and (f) saturated mass measurement.

## 4 Results and Discussion

### 4.1 Assessment of Chloride Penetration Profiles in Relation to the Concrete Cover Requirements of ABNT NBR 6118:2026

Figure 4 presents the average chloride penetration profiles obtained from the accelerated chloride migration test compared with the minimum concrete cover thicknesses recommended by the Brazilian standard ABNT NBR 6118:2026 for environmental exposure classes III (left) and IV (right) for slabs (refer to the black line) and beams, columns, and elements in contact with soil (refer to the orange line).

**Figure 4:**
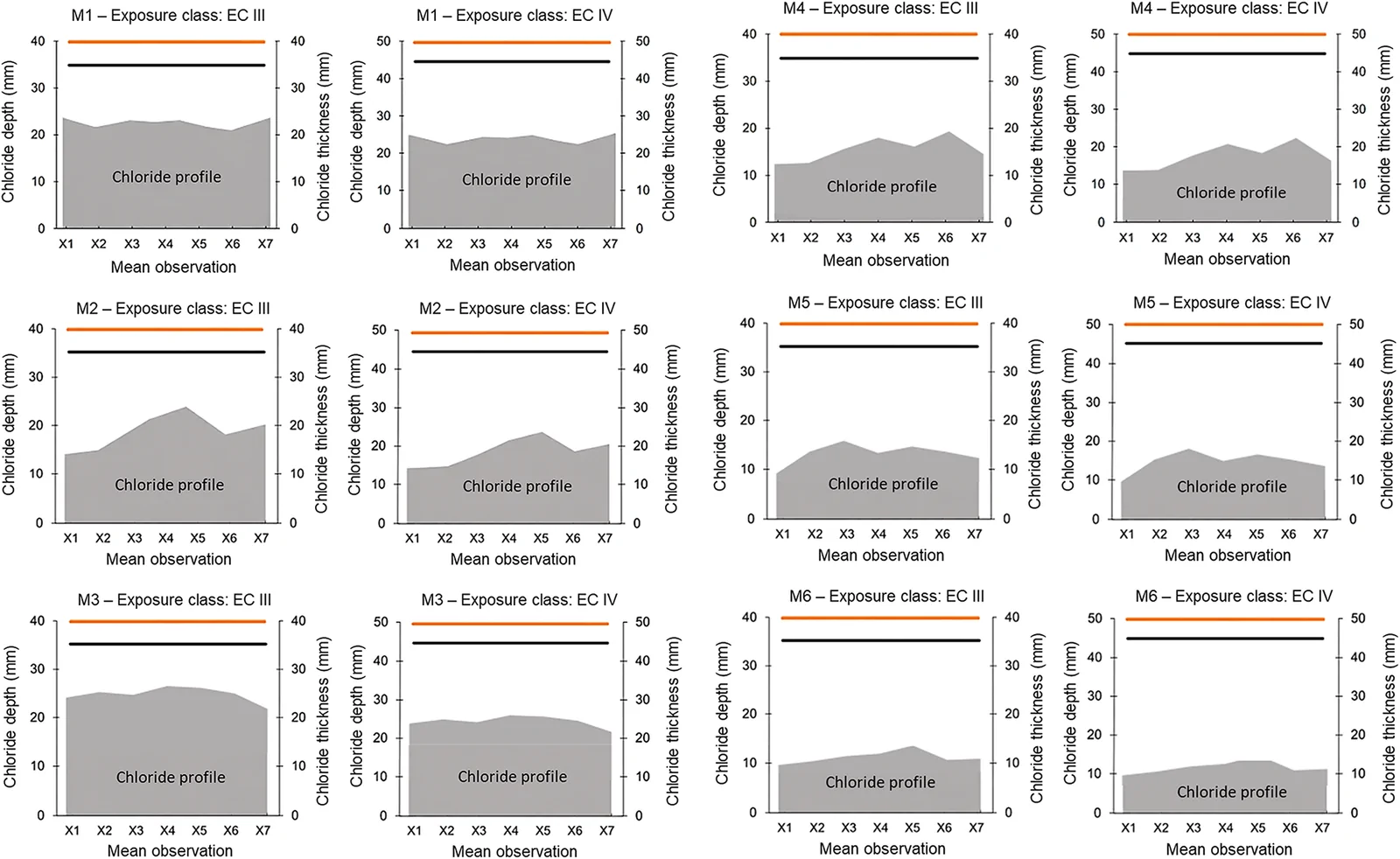
Comparison between the average chloride penetration profiles obtained from the accelerated chloride migration test and the minimum concrete cover thicknesses recommended by ABNT NBR 6118:2026 for exposure classes III and IV. The shaded regions represent the measured chloride penetration profiles for mixtures M1, M2, M3, M4, M5, and M6, while the horizontal reference lines indicate the minimum cover thicknesses prescribed for reinforced concrete structural elements under each environmental exposure condition for slabs (black line) and beams, columns, and elements in contact with soil (orange line).

As previously described, for reinforced concrete slabs, ABNT NBR 6118:2026 prescribes nominal concrete cover thicknesses of 35 mm and 45 mm for environmental exposure classes III and IV, respectively. For beams, columns, and elements in contact with soil, the required cover thicknesses increase to 40 mm and 50 mm, respectively, reflecting the greater severity of chloride exposure and the higher risk of reinforcement corrosion associated with these structural conditions.

The experimental results demonstrated that all investigated mixtures presented average chloride penetration depths lower than the minimum cover thicknesses prescribed by the Brazilian standards. This observation indicates that under the accelerated migration conditions adopted in this study, the normative cover thicknesses provided an effective physical barrier against chloride ingress, preventing the chloride front from reaching the reinforcement protection zone defined by the standard.

An important aspect of the results is that some mixtures exceeded the maximum water-to-cement ratio limits established by ABNT NBR 6118:2026 for aggressive exposure conditions, while still presenting chloride penetration depths below the prescribed concrete cover thicknesses. According to the Brazilian standard, maximum water-to-cement ratios of 0.55 and 0.45 are recommended for exposure classes III and IV, respectively. Consequently, mixtures M1 (0.68), M2 (0.63), and M3 (0.58) would not satisfy the requirements for exposure class III, whereas mixtures M1 to M5 (0.48) would theoretically be unsuitable for exposure class IV. Only mixture M6 (0.43) satisfied the maximum water-to-cement ratio constraint prescribed by the Brazilian standard for exposure class IV. Nevertheless, all mixtures remained below the normative coverage limits during the accelerated chloride migration test. These findings suggest that the prescriptive durability approach adopted by the Brazilian standards incorporates significant safety margins.

Table 6 summarizes the maximum average chloride penetration depth (*C*_d_) obtained for each concrete mixture, together with the percentage of chloride penetration relative to the minimum concrete cover thicknesses prescribed by ABNT NBR 6118:2026 for slabs (*C*_d_s__) and for beams, columns, and elements in contact with soil (*C*_d_b/c/s__) under environmental exposure classes III and IV. The percentage values for *C*_d_s__ and for *C*_d_b/c/s__ were calculated by relating the maximum average chloride penetration depth to the corresponding minimum concrete cover thickness (MCT) prescribed by ABNT NBR 6118:2026 for each environmental exposure class and structural element, according to eq 6. Minimum concrete cover thicknesses of 35 and 45 mm were considered for slabs under exposure classes III and IV, respectively, while values of 40 and 50 mm were adopted for beams, columns, and elements in contact with soil under exposure classes III and IV, respectively.

6 $$C_{d_{s}}=\frac{C_{d}}{\mathrm{MCT}}\cdot 100\%$$

**Table 6:** Maximum Average Chloride Penetration Depth (*C*_d_) and Percentage of Chloride Penetration Relative to the Minimum Concrete Cover Thicknesses Prescribed by ABNT NBR 6118:2023 for Slabs (*C*_d_s__) and for Beams, Columns, and Elements in Contact with Soil (*C*_d_b/c/s__) under Environmental Exposure Classes III and IV

Mixture	*C*_d_ (mm)	*C*_d_s__ (%) for EC III	*C*_d_s__ (%) for EC IV	*C*_d_b/c/s__ (%) for EC III	*C*_d_b/c/s__ (%) for EC IV
M1	25.00	71.43	55.56	62.50	50.00
M2	25.33	72.38	56.30	63.33	50.67
M3	25.17	71.90	55.93	62.92	50.33
M4	19.00	54.29	42.22	47.50	38.00
M5	15.67	44.76	34.81	39.17	31.33
M6	12.67	36.19	28.15	31.67	25.33

For slabs, the maximum chloride penetration percentages relative to the normative minimum concrete thickness varied from approximately ∼36% to ∼71% for exposure class III and from ∼28% to ∼56% for exposure class IV. For beams, columns, and elements in contact with soil, the percentages were naturally lower due to the greater cover thicknesses prescribed by the standard, ranging from approximately 25% to 63%.

These observations reinforce that the durability performance of reinforced concrete structures depends not only on concrete quality but also on the adequate execution and maintenance of the normative minimum cover thicknesses. Although ABNT NBR 6118 adopts relatively conservative recommendations due to the limited subdivision of environmental exposure classes, the results indicate that the prescribed cover thicknesses are effective in preventing chloride penetration from reaching the reinforcement under the investigated conditions.

It should be emphasized that the present analysis considers only chloride transport by migration under the accelerated laboratory conditions. Other deterioration mechanisms and field exposure conditions, such as cracking, carbonation, cyclic wetting and drying, temperature variations, and inadequate execution practices, were not considered within the scope of this study. Nevertheless, the results demonstrate the important role of the concrete cover thickness and matrix quality in enhancing the durability performance of reinforced concrete structures exposed to chloride-rich environments.

Although the comparison with the recommendations of ABNT NBR 6118:2026 provides an important practical assessment regarding the effectiveness of the prescribed durability requirements, the chloride penetration behavior observed among the investigated mixtures also suggests strong relationships between the chloride ingress resistance and the physical, mechanical, and transport-related properties of the concretes. Therefore, in order to better understand the influence of matrix densification and pore structure refinement on chloride transport behavior, the relationships among chloride penetration depth, non-steady-state chloride migration coefficient (*D*_ns_), compressive strength, void index, and ultrasonic pulse velocity (UPV) are discussed in the following section.

### 4.2 Correlation of Chloride Penetration Depth with *D*_ns_, Compressive Strength, Void Index, and UPV

Table 7 summarizes the results obtained for all investigated concrete mixtures (M1–M6), produced with water-to-cement ratios ranging from 0.43 to 0.68. The relationships among chloride penetration depth (*C*_d_), non-steady-state chloride migration coefficient (*D*_ns_), compressive strength, void index (VI), and ultrasonic pulse velocity (UPV) are presented in Figure 5 through linear regression analyses.

**Figure 5:**
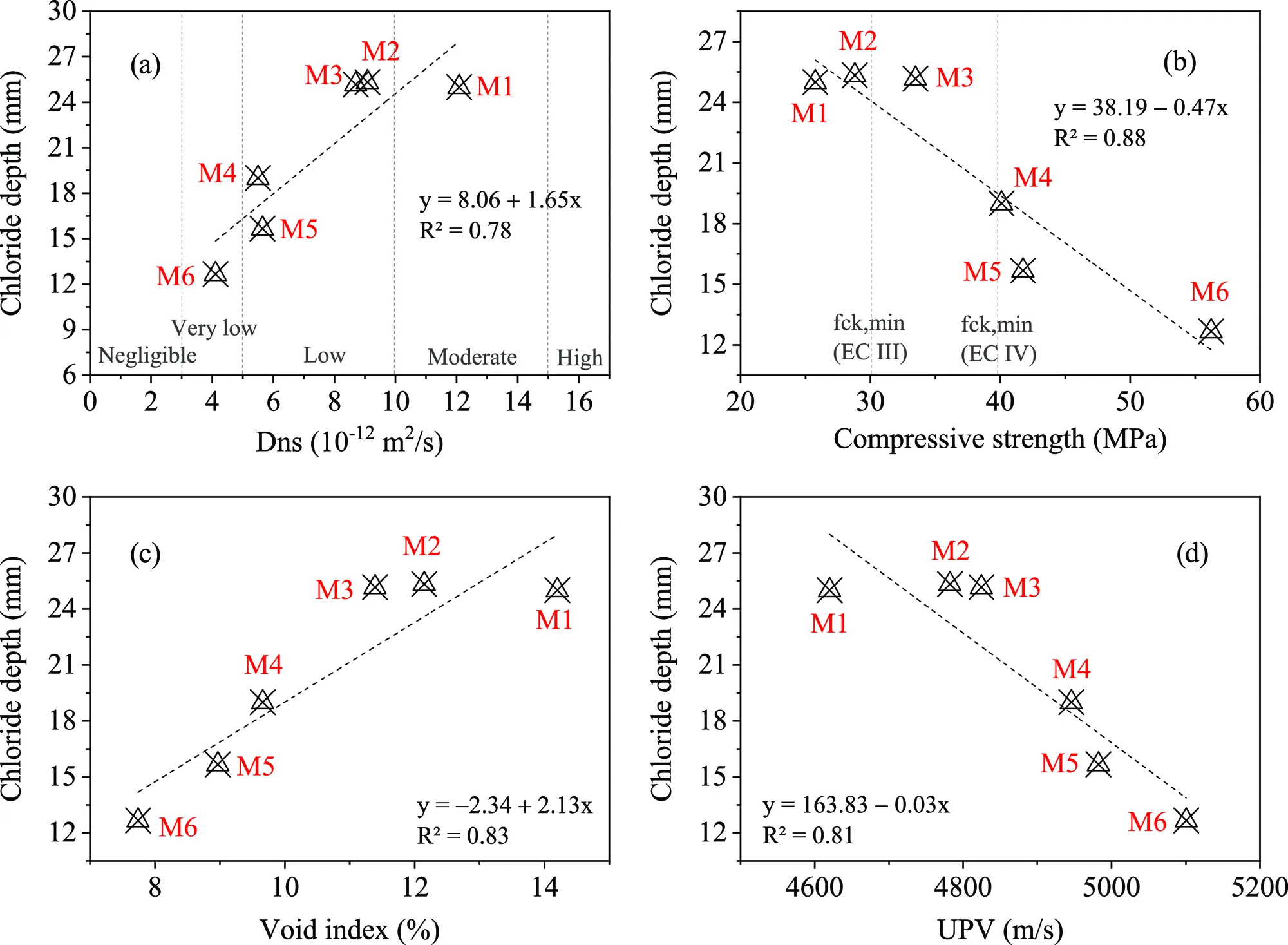
Linear regression analyses between chloride penetration depth (*C*_d_) and (a) non-steady-state chloride migration coefficient (*D*_ns_), (b) compressive strength, (c) void index (VI), and (d) ultrasonic pulse velocity (UPV) for the investigated concrete mixtures (M1–M6). The figure also presents the chloride permeability classifications proposed by Nilsson et al. based on *D*_ns_ values, as well as the minimum compressive strength requirements (fck,min) prescribed by ABNT NBR 6118:2026 for environmental exposure classes III and IV.

**Table 7:** Average Values of Chloride Penetration Depth (*C*_d_), Non-Steady-State Chloride Migration Coefficient (*D*_ns_), Compressive Strength, Void Index (VI), and Ultrasonic Pulse Velocity (UPV) for the Investigated Concrete Mixtures Produced with Different Water-to-Cement Ratios

Mixture	w/c ratio	*C*_d_ (mm)	*D*_ns_ (10^–12^ m^2^/s)	Compressive strength (MPa)	VI (%)	UPV (m/s)
M1	0.68	25.00	12.08	25.76	14.20	4620.1
M2	0.63	25.33	9.08	28.79	12.15	4782.2
M3	0.58	25.17	8.70	33.47	11.39	4824.6
M4	0.53	19.00	5.50	40.10	9.66	4945.8
M5	0.48	15.67	5.65	41.76	8.97	4982.3
M6	0.43	12.67	4.11	56.25	7.74	5100.8

As expected, the progressive reduction in the non-steady-state chloride migration coefficient (*D*_ns_) from mixtures M1 to M6 was accompanied by a corresponding decrease in chloride penetration depth, indicating an improvement in resistance to chloride transport through matrix densification and pore structure refinement (Figure 5a). Although the NT Build 492 methodology used to determine *D*_ns_ does not establish permeability classifications, Nilsson et al. proposed reference ranges for concretes at 28 days based on chloride migration coefficient values. According to this classification, concretes presenting *D*_ns_ values higher than 15 × 10^–12^ m^2^/s are considered to exhibit high chloride permeability; values between 10 and 15 × 10^–12^ m^2^/s indicate moderate permeability; values between 5 and 10 × 10^–12^ m^2^/s correspond to low permeability; values between 2.5 and 5 × 10^–12^ m^2^/s indicate very low permeability; and values lower than 2.5 × 10^–12^ m^2^/s are considered negligible. Based on these criteria, mixture M1 may be classified as presenting moderate chloride permeability, mixtures M2 to M5 as low permeability concretes, and mixture M6 as exhibiting very low chloride permeability.

The progressive reduction in chloride penetration depth with increasing compressive strength further demonstrates that the improvement in durability performance was not solely associated with a mechanical gain, but rather with the development of a denser and less interconnected pore structure (Figure 5b). Even mixtures M1 and M2, which did not achieve the minimum compressive strength of 30 MPa prescribed by ABNT NBR 6118:2026 for environmental exposure class III, exhibited chloride penetration depths corresponding to approximately 72% of the minimum concrete cover thickness required by the standard. In contrast, mixtures M5 and M6, which satisfied the minimum compressive strength requirements for class IV exposure conditions, presented chloride penetration depths ranging between approximately 28% and 45% of the prescribed cover thickness. These results suggest that the increase in compressive strength produced a disproportionately larger improvement in chloride transport resistance than would be expected from strength gain alone, highlighting the role of pore refinement and reduced capillary connectivity in limiting the ionic ingress.

Although the compressive strength is not a direct transport-related parameter, the strong correlation observed among the compressive strength, chloride penetration depth, and *D*_ns_ indicates that the mechanisms controlling mechanical performance and chloride transport resistance evolved simultaneously as the water-to-cement ratio decreased. Among the evaluated parameters, the compressive strength presented one of the highest coefficients of determination with chloride penetration depth, reinforcing its suitability as an indirect durability indicator under chloride-rich exposure conditions. This observation provides important experimental support for the durability philosophy adopted by ABNT NBR 6118:2026, which uses minimum compressive strength requirements as simplified prescriptive parameters to indirectly control permeability and transport resistance.

The relationship between the chloride penetration depth and void index further reinforces the central role of pore structure characteristics on chloride transport behavior (Figure 5c). Concrete presenting higher void indices exhibited greater chloride penetration depths, indicating that differences in the volume and continuity of voids are associated with chloride ingress. The reduction in void index observed as the water-to-cement ratio decreased is consistent with the general understanding that lower water availability can result in a denser cementitious matrix and reduced transport pathways.^5,9,29^ However, direct pore structure characterization was not performed in the present study. Therefore, the observed reduction in the void index and chloride penetration should be interpreted as indirect evidence of changes in concrete compactness and transport behavior rather than direct experimental confirmation of pore refinement or changes in pore connectivity. Such characterization would require complementary techniques, such as mercury intrusion porosimetry, nitrogen adsorption, micro-CT, or microscopic analysis, and should be considered in future investigations.

The progressive reduction in the void index observed from M1 to M6 reflects the gradual refinement of the capillary network promoted by lower water availability during concrete production. This behavior is consistent with classical transport theory, in which reductions in capillary porosity and pore interconnectivity significantly decrease permeability, diffusivity, and ionic mobility within the hardened matrix.^5,29^ Consequently, the results help explain the rationale behind the increasingly restrictive durability requirements prescribed by ABNT NBR 6118:2026 for more aggressive environmental exposure classes.

In addition to destructive transport-related tests, ultrasonic pulse velocity (UPV) measurements were also performed as a nondestructive technique to evaluate internal concrete quality (Figure 5d). The inverse relationship observed between UPV and chloride penetration depth demonstrates that concrete with higher wave propagation velocities tended to present lower chloride ingress. Since ultrasonic propagation is strongly influenced by internal discontinuities, pore distribution, and microstructural heterogeneity, the results indicate that UPV may provide a useful indirect assessment of transport resistance and durability performance, which is consistent with the findings of Jacobsen et al.,^9^ who observed that reductions in UPV caused by internal cracking were accompanied by substantial increases in chloride migration rates. The progressive increase in UPV from M1 to M6 suggests the development of increasingly homogeneous and compact internal structures, which is consistent with the simultaneous reductions observed in the void index, *D*_ns_, and chloride penetration depth.

Finally, the comparison between the chloride penetration depth and the minimum concrete cover thicknesses prescribed by ABNT NBR 6118:2026 demonstrated the conservative nature of the Brazilian durability design approach. Even the mixtures produced with the highest water-to-cement ratios and lower compressive strengths did not exceed the normative cover thicknesses recommended for environmental exposure classes III and IV under the accelerated migration conditions adopted in this study. Nevertheless, the substantial differences observed among the investigated mixtures reinforce that compliance with minimum compressive strength requirements alone may not fully represent chloride transport resistance, particularly when concrete presents distinct pore structures and permeability characteristics.

Overall, all evaluated parameters, including *D*_ns_, compressive strength, void index, and UPV, exhibited strong correlations with the chloride penetration depth, demonstrating their sensitivity to changes in pore structure refinement, matrix densification, and transport resistance. Although these properties are not direct measurements of chloride ingress itself, the consistent relationships observed indicate that they may serve as reliable indirect indicators for assessing the susceptibility of concrete to chloride penetration. Consequently, the combined interpretation of mechanical, physical, transport-related, and nondestructive parameters may provide a more comprehensive evaluation of concrete durability performance under chloride-rich exposure conditions.

Rissardi et al.^30^ investigated the resistance of concrete mixtures designed according to the durability requirements of ABNT NBR 6118 against chloride attack under accelerated exposure conditions. The study focused on concretes corresponding to environmental exposure classes III and IV, produced with maximum water-to-cement ratios of 0.55 and 0.45, respectively, according to the recommendations of ABNT NBR 6118 and NBR 12655. Although compressive strength was not experimentally determined, the mixtures were proportioned to satisfy the minimum strength classes prescribed by the Brazilian standard, namely C30 and C40 for classes III and IV, respectively. The chloride attack was evaluated through an accelerated salt spray exposure test based on ASTM B117. In this procedure, the specimens were exposed inside a chamber maintained at 35 °C under continuous spraying of a 5% NaCl solution with a pH between 6.5 and 7.2 for 1728 h (72 days). After exposure, the specimens were longitudinally split and sprayed with AgNO_3_ solution in order to reveal the chloride penetration front by colorimetric identification. The chloride penetration depth was determined through transverse measurements performed at predefined positions along the specimen section.

The results demonstrated a clear reduction in chloride penetration depth with a decreasing water-to-cement ratio. For the concrete produced with a w/c ratio of 0.55 (class III), the average transverse chloride penetration depth was approximately 3.1 mm, whereas the concrete produced with a w/c ratio of 0.45 (class IV) presented an average penetration depth close to 1.1 mm. The corresponding maximum transverse penetration depths were approximately 8.3 mm and 3.7 mm for classes III and IV, respectively. An important aspect highlighted by the authors concerns the substantial difference between average and maximum chloride penetration depths, indicating that localized chloride ingress may be significantly greater than the average values commonly used in conventional analyses. According to the authors, this heterogeneous penetration behavior may contribute to localized reinforcement depassivation and the formation of corrosion pits, even when the average chloride penetration depth remains relatively low. However, despite the localized increase in chloride penetration, the maximum depths reported by the authors remained considerably lower than the minimum nominal concrete cover thicknesses prescribed by ABNT NBR 6118 for environmental exposure classes III and IV, which range from 35 to 50 mm, depending on the structural element.

Although the methodology was effective in differentiating the chloride resistance of concretes produced with different water-to-cement ratios, the penetration depths obtained remained relatively limited, even after 72 days of continuous exposure. This observation suggests that the main limitation of the ASTM B117 salt spray methodology is associated with the relatively slow chloride transport kinetics under diffusion and absorption mechanisms, requiring long exposure periods to produce chloride penetration fronts representative of long-term field exposure conditions. Nevertheless, the results clearly reinforce the influence of the water-to-cement ratio and matrix densification on chloride ingress resistance.

Since the present study focused exclusively on SCM-free concretes produced with varying water-to-cement ratios, the influence of supplementary cementitious materials on the chloride transport behavior was not directly investigated within the scope of this work. Nevertheless, the literature provides important insights regarding the effect of the binder composition on chloride penetration resistance. De Jesus et al.^25^ evaluated chloride penetration depth and non-steady-state chloride migration coefficients (*D*_ns_) using the NT Build 492 methodology and observed substantially improved chloride resistance for concretes produced with high-SCM-content cements, particularly PC III and PC IV binders. Figure 6 compares the relationship between chloride penetration depth and *D*_ns_ obtained in the present work with the results reported by De Jesus et al.,^25^ highlighting the strong dependence of chloride transport behavior on both water-to-cement ratio and binder composition.

**Figure 6:**
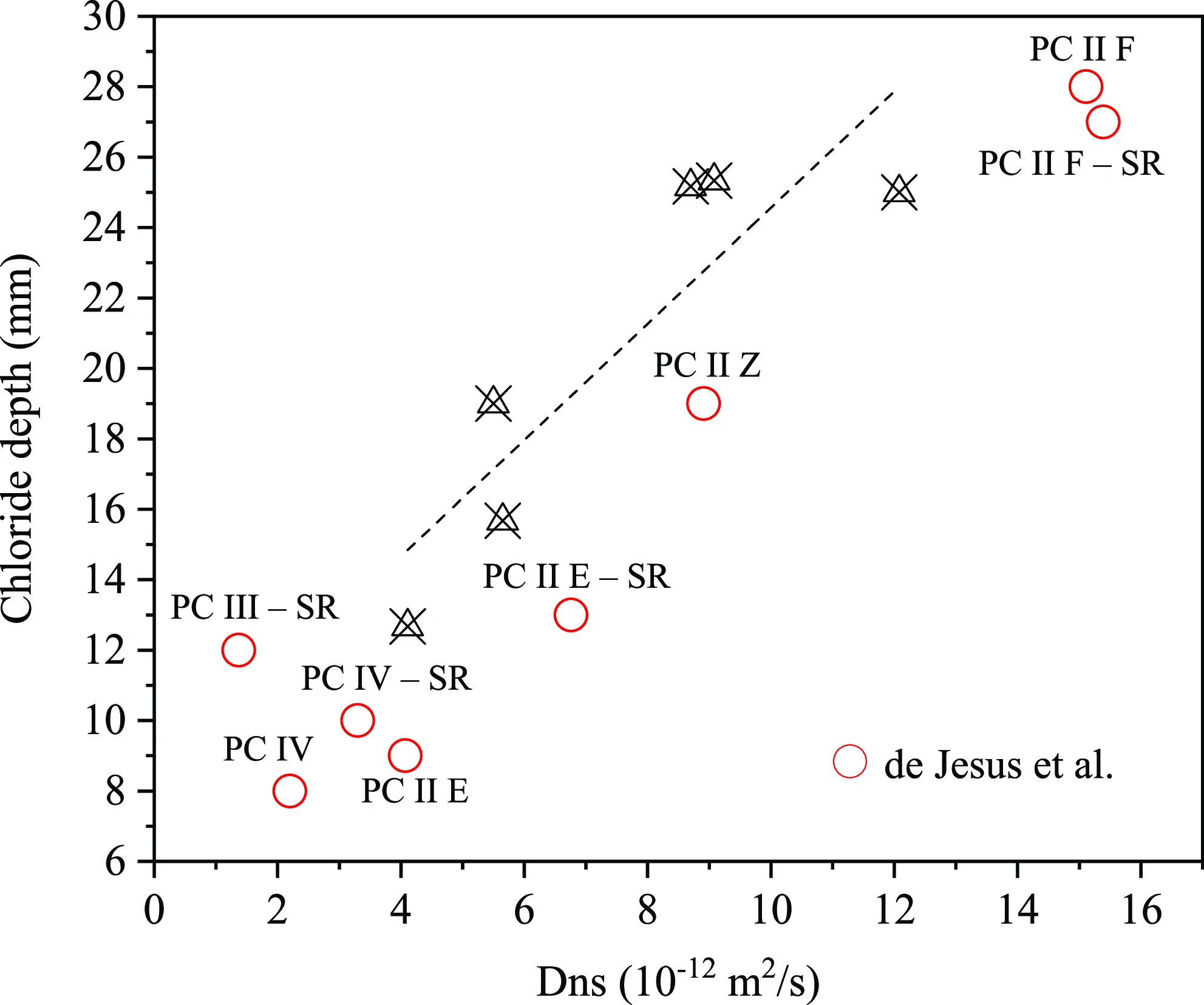
Chloride depth against *D*_ns_ values of this work compared to literature data.

These systems presented significantly lower chloride penetration depths and reduced *D*_ns_ values, indicating enhanced resistance to ionic transport due to pore structure refinement and increased tortuosity promoted by slag and pozzolanic reactions. In contrast, filler-rich concretes based on PC II F cement exhibited the highest chloride penetration depths and the largest *D*_ns_ values among the investigated mixtures, demonstrating a considerably greater susceptibility to chloride ingress. These observations reinforce that chloride transport resistance is governed not only by the water-to-cement ratio but also by the long-term evolution of the cementitious matrix and binder composition. Consequently, evaluating chloride migration coefficients and their time dependency becomes essential for predicting the long-term durability performance of reinforced concrete structures exposed to chloride-rich environments, as discussed in the following section on service-life estimation.

### 4.3 Service-Life Estimation

The Brazilian standard ABNT NBR 6118:2026, together with ABNT NBR 15575, is designed and calibrated to provide a minimum design service life of approximately 50 years for reinforced concrete structures when adequate execution, curing, use, and maintenance conditions are ensured. In order to achieve this durability target, the Brazilian normative approach adopts prescriptive requirements based on environmental exposure classes, minimum compressive strength, maximum water-to-cement ratio, minimum cement consumption, and nominal concrete cover thickness. The fundamental concept behind this approach is that increasingly aggressive exposure conditions require progressively denser and less permeable concretes combined with larger concrete cover thicknesses in order to delay reinforcement depassivation and chloride-induced corrosion initiation.

Despite the widespread use of prescriptive durability approaches in structural design standards, several methodologies have been proposed for estimating the service life of reinforced concrete structures exposed to chloride-rich environments. In general, these methodologies aim to estimate the time required for chlorides to reach the reinforcement level at concentrations that are capable of initiating corrosion. The most common approaches include empirical models based on accelerated durability tests, semiempirical transport models, probabilistic analyses considering environmental and material uncertainties, and mechanistic diffusion-based models derived from chloride transport theory.

An important aspect demonstrated by the present results is that the chloride penetration depth obtained during accelerated testing does not necessarily provide a direct estimation of the long-term service life of the reinforced concrete structures. Although all investigated mixtures presented chloride penetration depths lower than the minimum concrete cover thicknesses prescribed by ABNT NBR 6118:2026 for environmental exposure classes III and IV, the estimated service-life performance may still vary substantially among the mixtures due to differences in chloride transport kinetics. In other words, the experimental chloride penetration depth represents only the chloride advancement reached during a specific exposure period, whereas the long-term durability depends on the future rate of chloride transport through the concrete matrix over decades of exposure. Consequently, concretes presenting similar chloride penetration depths during accelerated tests may still exhibit significantly different long-term performances, depending on their pore structure refinement, permeability, ionic diffusivity, and aging behavior.

Among the available methodologies for chloride-induced service-life estimation, diffusion-based approaches derived from Fick’s second law are currently the most widely employed in durability engineering due to their relatively strong physical basis and practical applicability. These models describe chloride transport through the concrete matrix as a diffusion-controlled process governed by concentration gradients between the external environment and the concrete pore solution. The classical solution for one-dimensional chloride transport is expressed in eq 7, where *C*_crit_ is the critical chloride concentration at depth *x* and exposure time *t*, *C*_s_ is the surface chloride concentration, and *D*_a_(*t*) is the time-dependent apparent diffusion coefficient. According to this approach, corrosion initiation is assumed to occur when the chloride concentration at the reinforcement depth reaches a critical chloride threshold (*C*_crit_) capable of depassivating the steel surface.

7 $$C_{\left(x,t\right)}=C_{\mathrm{crit}}=C_{s}\left[1-\mathrm{erf}\left(\frac{x}{2\sqrt{D_{a}\left(t\right)\times t}}\right)\right]$$

Since chloride diffusivity decreases over time due to continued hydration and progressive pore refinement, time-dependent diffusion models often incorporate an aging factor, expressed in eq 8, where *D*_ref_ is the reference chloride diffusion coefficient, which at time *t*_ref_ is the reference age, and *m* is the aging factor associated with the progressive reduction in chloride diffusivity over time.

8 $$D_{a}\left(t\right)=D_{\mathrm{ref}}{\left(\frac{t_{\mathrm{ref}}}{t}\right)}^{m}$$

From eqs 7 and 8, service life can be obtained analytically from eq 9.


9 $$t={\left[\frac{x^{2}}{4{\left({\mathrm{erf}}^{-1}\left(1-\frac{C_{\mathrm{crit}}}{C_{s}}\right)\right)}^{2}D_{\mathrm{ref}}\times t_{\mathrm{ref}}^{m}}\right]}^{1/\left(1-m\right)}$$

The service-life prediction obtained from Fick-based models is strongly influenced by several parameters associated with both material characteristics and environmental exposure conditions. Among the most important factors are the apparent chloride diffusion coefficient, the surface chloride concentration, the critical chloride threshold for reinforcement depassivation, the concrete cover thickness, and the aging factor associated with the progressive reduction in chloride diffusivity over time due to continued cement hydration and pore refinement. Additional variables such as temperature, moisture fluctuations, wetting and drying cycles, cracking, chloride binding capacity, and environmental exposure severity may also significantly affect long-term chloride transport behavior.^5,9,29^

In this context, the apparent chloride diffusion coefficient plays a particularly important role because it directly controls the rate of chloride transport through the cementitious matrix. Concrete presenting lower diffusion or migration coefficients generally exhibits slower chloride advancement and consequently longer estimated service lives. This explains why mixtures with similar experimental chloride penetration depths may still present substantially different predicted service-life performances. Concretes with high chloride transport resistance tend to exhibit a progressive reduction in chloride ingress rate over time due to continued matrix densification, whereas concretes with more connected pore structures may maintain relatively high transport kinetics throughout their service life.^5,29^

Therefore, although the comparison between experimental chloride penetration depth and normative concrete cover thickness provides an important practical durability assessment, transport-related parameters such as the non-steady-state chloride migration coefficient (*D*_ns_) allow a more refined evaluation of long-term chloride-induced deterioration. Consequently, combining accelerated chloride migration tests with diffusion-based service-life modeling may provide a more representative interpretation of the durability performance of reinforced concrete structures exposed to chloride-rich environments.

Additionally, it should be emphasized that the apparent diffusion coefficient adopted in Fick-based service-life models is not strictly equivalent to the non-steady-state chloride migration coefficient (*D*_ns_) obtained from NT Build 492. While *D*_ns_ is determined under the influence of an externally applied electrical field, Fick-based models describe natural chloride transport governed by concentration gradients. Consequently, migration coefficients are generally higher than natural diffusion coefficients due to the acceleration of ionic transport during the migration test. Thus, the *D*_ref_ was estimated from the experimental *D*_ns_ using the empirical correlation *D*_ns_ = 0.5 × D_ref_. This conversion factor is widely adopted in comparative service-life frameworks to align accelerated electrical migration data with long-term natural diffusion transport mechanisms under field conditions, consistent with guidelines from fib Bulletin 34 and regional calibrations for plain Portland cement matrices.^31^

Although this simplification does not fully represent the complexity of chloride transport under natural exposure conditions, it allows for the use of experimentally obtained migration coefficients as comparative indicators of long-term chloride resistance. Therefore, the present service-life estimations should be interpreted primarily as comparative durability indicators rather than exact lifetime predictions. Even so, the use of *D*_ns_-derived diffusion coefficients provides valuable information regarding the relative chloride transport resistance and long-term durability potential of the investigated concrete.

In this sense, the present study adopted the experimentally obtained (*D*_ns_) values and the minimum concrete cover thicknesses prescribed by ABNT NBR 6118:2026 for exposure classes III and IV to estimate the service life of concretes exposed to different chloride-rich environments, including marine immersion zones, splash and tidal regions, airborne marine exposure, and coastal atmospheric conditions at different distances from the shoreline. Additional parameters required for the service-life calculations were adopted based on values commonly reported in the literature and on probabilistic durability studies available for reinforced concrete structures under marine exposure.

The aging factor (*m*), which represents the progressive reduction in chloride diffusivity over time due to continued hydration and pore structure refinement, was assumed to be 0.4 and 0.3 in the present analysis. These two values were specifically tested to evaluate the sensitivity of the service-life predictions to the aging exponent. The adoption of *m* = 0.4 is consistent with the probabilistic durability analyses performed by Wally et al.,^32^ who used a normal distribution with a mean value of 0.4 for chloride transport modeling in marine environments using Duracon, fib Model Code, and Life-365 approaches. This value is also frequently reported in service-life modeling studies involving conventional Portland cement concretes exposed to chloride-rich environments. Several authors have experimentally demonstrated that the aging factor is strongly influenced by the concrete composition, hydration kinetics, capillary porosity refinement, and the presence of supplementary cementitious materials (SCMs). In general, concretes exhibiting stronger long-term microstructural densification tend to present higher aging factors. For plain Portland cement concretes, Audenaert et al.^33^ reported aging factors ranging from 0.23 to 0.40 for CEM I 42.5 R-based concretes with water-to-cement ratios between 0.35 and 0.50. Frederiksen et al.^34^ reported values between approximately 0.4 and 0.6 for concretes with w/c ratios varying from 0.4 to 0.7. On the other hand, Lars-Olof^27^ and Dhandapani and Santhanam^35^ suggested a fixed value of 0.3 for ordinary concretes with w/c ratios lower than 0.6. Similarly, Mangat and Molloy^36^ reported aging factors close to 0.42 and 0.46 for plain concretes with w/c ratios between 0.4 and 0.6.

Experimentally, the aging factor can be determined by measuring the apparent chloride diffusion or migration coefficient at different curing ages and fitting the results to a time-dependent power law relationship. In practice, accelerated migration tests or natural diffusion tests are commonly performed at different ages, such as 28, 56, 90, and 180 days, and the obtained coefficients are plotted as a function of time on a logarithmic scale. The slope of the fitted regression line corresponds to the aging factor m. Consequently, concrete mixtures exhibiting greater long-term pore refinement and continued hydration generally present higher aging factors and more substantial reductions in chloride diffusivity throughout their service life. This work adopted an aging factor of 0.4.

The adopted surface chloride concentration (*C*_s_) values were selected according to the severity of the exposure environment, considering the literature data reported for marine structures subjected to different chloride exposure conditions. Wally et al.^32^ discussed that *C*_s_ is strongly influenced by factors such as seawater proximity, wind intensity, wetting-drying cycles, rainfall, and exposure duration. The authors also highlighted the substantial variability associated with *C*_s_, especially under marine exposure, and reported representative values obtained for different coastal environments in Brazil. Surface chloride concentrations around 3.0–3.5% are commonly associated with highly aggressive splash and tidal zones, whereas lower values between approximately 0.6 and 1.1% are generally observed in airborne marine environments located further from the coastline. On the basis of these observations, the present study adopted distinct Cs values according to the assumed exposure condition in order to provide a more realistic representation of environmental aggressiveness.

The critical chloride threshold for steel depassivation (*C*_crit_) was assumed as 0.4% by mass of cement, which is one of the most widely adopted values in durability and service-life modeling studies for reinforced concrete structures exposed to chlorides. Although the literature reports considerable variability in *C*_crit_ due to differences in materials, exposure conditions, testing methodologies, and corrosion detection criteria,^37^ values close to 0.4% by weight of cement have been widely adopted in engineering practice and durability-oriented design approaches in both European and North American contexts. Angst et al.^38^ reported that tolerable chloride contents are commonly limited to approximately 0.4% by weight of cement in practical applications, while Wally et al.^32^ highlighted that this value remains one of the most frequently employed assumptions in service-life analyses despite the inherent stochastic nature and uncertainty associated with chloride threshold values.

It should also be emphasized that supplementary cementitious materials may significantly alter both the apparent chloride diffusion coefficient and the long-term evolution of the chloride transport properties. Pozzolanic materials and latent hydraulic additions generally reduce pore connectivity and increase chloride binding capacity, resulting in lower effective diffusivities and enhanced long-term durability performance.^5^ Consequently, mixtures containing supplementary cementitious materials often present higher aging factors and lower chloride penetration rates compared with plain Portland cement systems.

Therefore, although the present service-life estimations are based on simplified assumptions and should primarily be interpreted as comparative durability indicators rather than exact predictions of structural lifetime, the adopted methodology provides a physically consistent framework for comparing the chloride transport resistance and long-term durability potential of the investigated concretes under different marine exposure conditions. As emphasized by Wally et al.,^32^ service-life prediction models should not be interpreted as absolute estimations, since chloride ingress is influenced by numerous environmental and material uncertainties. Instead, such models should be understood as valuable engineering tools for comparative durability assessment, long-term performance analysis, and maintenance planning.

Table 8 summarizes the parameters and assumptions adopted for the chloride ingress-based service-life prediction model. The selected values were defined based on literature recommendations and commonly accepted durability modeling approaches for reinforced concrete exposed to marine environments. Table 9 presents the estimated service life to chloride-induced corrosion initiation for reinforced concrete structures exposed to different marine conditions. The predictions were obtained using the time-dependent form of Fick’s second law, considering the experimentally determined non-steady-state migration coefficients (*D*_ns_) at 28 days for mixtures M1 to M6, together with literature-based assumptions for the aging factor and critical chloride threshold. Values exceeding 100 years are reported as >100 years because the uncertainty associated with the adopted transport and environmental assumptions does not justify greater numerical precision.

**Table 8:** Parameters and Assumptions Adopted for Chloride Ingress-Based Service-Life Prediction of Reinforced Concrete Structures under Marine Exposure Conditions

Parameter	Symbol	Assumed Value (s)	Basis/Justification
Apparent chloride diffusion coefficient	(*D*_ref_)	*D*_ref_ = 0.5 × *D*_ns_	Simplified empirical conversion frequently adopted in service-life analyses
Aging factor	(*m*)	0.4 and 0.3	Literature-based value for probabilistic chloride transport modeling
Reference time	(*t*_ref_)	28 days	Time at which the *D*_ns_ was measured
Surface chloride concentration–splash/tidal zone	(*C*_s_)	3.0–3.5%	Literature-based value for highly aggressive marine exposure conditions
Surface chloride concentration–marine atmosphere near coastline	(*C*_s_)	1.0–2.0%	Literature-based value for moderate airborne chloride exposure
Surface chloride concentration–distant coastal atmosphere	(*C*_s_)	0.6–1.1%	Literature-based value for lower chloride accumulation at greater distances from shoreline
Critical chloride threshold	(*C*_crit_)	0.4% by mass of cement	Widely adopted depassivation threshold in durability modeling

**Table 9:** Estimated Service Life (years) to Chloride-Induced Corrosion Initiation for Reinforced Concrete Structures, Calculated Using the Time-Dependent Form of Fick’s Second Law^a^

		Estimate of service life (years)					
Surface chloride (*C*_s_)	Environmental exposure classes (EC) adopted to determine the minimum concrete cover thickness	M1	M2	M3	M4	M5	M6
Splash/tidal zone (3.25%)	EC III–slab (35 mm)	9.2	14.7	15.8	34.0	32.5	55.2
	EC III–beams, columns, and elements in contact with soil (40 mm)	14.3	23.0	24.7	53.1	50.7	86.2
	EC IV–slab (45 mm)	21.2	34.1	36.6	78.6	75.1	>100
	EC IV–beams, columns, and elements in contact with soil (50 mm)	30.1	48.4	52.0	>100	>100	>100
Marine atmosphere near coastline (1.5%)	EC III–slab (35 mm)	27.3	44.0	47.2	∼100	97.0	>100
	EC III–beams, columns, and elements in contact with soil (40 mm)	42.7	68.7	73.7	>100	>100	>100
	EC IV–slab (45 mm)	63.2	>100	>100	>100	>100	>100
	EC IV–beams, columns, and elements in contact with soil (50 mm)	89.8	>100	>100	>100	>100	>100
Distant coastal atmosphere (0.85%)	EC III–slab (35 mm)	>100	>100	>100	>100	>100	>100
	EC III–beams, columns, and elements in contact with soil (40 mm)	>100	>100	>100	>100	>100	>100
	EC IV–slab (45 mm)	>100	>100	>100	>100	>100	>100
	EC IV–beams, columns, and elements in contact with soil (50 mm)	>100	>100	>100	>100	>100	>100

a Results consider an aging factor *m* = 0.4, critical chloride threshold *C*_crit_ = 0.4%, and non-steady-state migration coefficients (*D*_ns_) at 28 days corresponding to concrete mixes M1 to M6; service lives are presented for different surface chloride contents (average) and exposure classes.

Among all investigated variables, the apparent chloride transport resistance represented by the *D*_ns_ values exerted one of the most significant influences on the estimated service life. Although the reduction in *D*_ns_ from M1 to M6 may initially appear moderate, the corresponding increase in predicted service life was highly amplified due to the nonlinear nature of chloride diffusion phenomena described by Fick-based models. This behavior demonstrates that relatively small improvements in chloride transport resistance may produce disproportionately large increases in the long-term durability performance. For instance, under splash/tidal exposure conditions and considering EC IV for beams, columns, and elements in contact with soil, the predicted service life increased from approximately 30 years for M1 to more than 100 years for M6, when considering an aging factor of *m* = 0.4, corresponding to an increase of approximately six times. This result highlights the strong sensitivity of service-life modeling to the apparent diffusion coefficient adopted in the analysis.

The influence of the environmental chloride concentration is also particularly significant. The highest surface chloride concentration, corresponding to splash/tidal zone exposure (*C*_s_ = 3.25%), produced the lowest estimated service-life values for all mixtures due to the greater chloride concentration gradient between the external environment and the concrete pore solution. Under these highly aggressive conditions, mixtures M1, M2, and M3 presented predicted service lives lower than the minimum design service life of 50 years established by the Brazilian durability framework for several exposure scenarios, especially for EC III conditions. Even mixtures M4 and M5 exhibited service lives below 50 years under some splash and tidal exposure scenarios when smaller cover thicknesses were considered. Only mixture M6 consistently achieved service lives above 50 years under all splash and tidal conditions when *m* = 0.4 was adopted.

In contrast, when the surface chloride concentration decreased to values representative of the marine atmosphere near the coastline (*C*_s_ = 1.5%) and distant coastal atmosphere (*C*_s_ = 0.85%), the predicted service-life values increased dramatically for all mixtures. Under distant coastal atmospheric exposure, all mixtures exhibited service lives above 100 years for several exposure conditions when *m* = 0.4 was considered, particularly for larger cover thicknesses. This behavior confirms that environmental exposure severity is one of the governing parameters controlling chloride ingress and reinforcement depassivation.

The effect of concrete cover thickness was equally expressive. Increasing the nominal cover thickness from 35 mm to 50 mm produced substantial increases in predicted service life for all investigated mixtures and exposure conditions. This behavior is directly associated with the quadratic dependence between chloride penetration distance and diffusion time predicted by Fick’s second law. Consequently, relatively small increases in cover thickness may significantly delay chloride arrival at the reinforcement depth. For example, under splash/tidal exposure and considering mixture M4 with *m* = 0.4, increasing the cover thickness from 35 mm to 50 mm increased the predicted service life from approximately 34 years to more than 100 years. This observation strongly supports the durability philosophy adopted by ABNT NBR 6118:2026, which prescribes progressively larger concrete cover thicknesses as environmental aggressiveness increases.

An important aspect evidenced by the results is that compliance with the minimum concrete cover thickness requirements of ABNT NBR 6118:2026 does not necessarily guarantee an equivalent long-term durability performance among different concretes. Although all investigated mixtures presented chloride penetration depths lower than the prescribed cover thicknesses during the accelerated migration tests, the estimated service-life values varied substantially depending on the transport properties of the concrete. This demonstrates that the experimental chloride penetration depth obtained during short-term accelerated testing represents only the instantaneous advancement of chlorides under a specific exposure period, whereas long-term durability depends primarily on the future chloride transport kinetics throughout decades of service exposure.

The results further indicate that mixtures M1–M3, which presented higher water-to-cement ratios, higher void indices, and higher chloride migration coefficients, were considerably more sensitive to severe chloride exposure conditions. Under splash/tidal exposure conditions, these mixtures frequently exhibited estimated service lives well below the minimum design service life commonly associated with Brazilian durability standards, particularly under EC III conditions. Conversely, mixtures M4–M6 exhibited substantially greater resistance to chloride ingress due to their denser microstructures and lower ionic transport capacity. Particularly for M6, the predicted service-life values exceeded 100 years under several exposure scenarios, demonstrating the strong beneficial effect of matrix refinement on chloride-induced durability performance.

Another important observation is that the predicted service-life values increased exponentially as the surface chloride concentration decreased from splash/tidal exposure conditions toward atmospheric marine environments. This demonstrates that environmental classification alone may not fully represent the actual chloride exposure severity experienced by a structure, since local chloride accumulation conditions may vary considerably depending on distance from the coastline, orientation, wetting and drying cycles, and microclimatic effects. Consequently, realistic estimations of the surface chloride concentration are fundamental for reliable service-life prediction.

It should also be emphasized that the present service-life estimations correspond only to the corrosion-initiation period associated with chloride ingress until reinforcement depassivation. The propagation stage of corrosion, including crack formation, steel section reduction, and structural deterioration after corrosion initiation, was not considered in the present analysis. Furthermore, the adopted Fick-based modeling assumes homogeneous concrete properties and diffusion-controlled chloride transport, while real structures may also be affected by cracking, carbonation, cyclic exposure conditions, temperature variations, moisture fluctuations, chloride binding effects, and execution deficiencies.^9^

Nevertheless, despite these limitations, the obtained results clearly demonstrate the strong influence of chloride transport resistance, environmental exposure severity, and concrete cover thickness on the long-term durability performance of the reinforced concrete structures. However, the large values obtained under less aggressive exposure conditions should not be interpreted as definitive structural service lives but rather as model-based estimates indicating lower relative chloride transport susceptibility under the adopted assumptions. The results further reinforce that transport-related durability parameters such as *D*_ns_ provide a more refined evaluation of chloride-induced deterioration potential than compressive strength alone, supporting the importance of combining accelerated chloride migration tests with service-life modeling approaches in durability-oriented concrete design.

Table 10 presents the estimated service-life values for chloride-induced corrosion initiation calculated using the time-dependent form of Fick’s second law, considering an aging factor *m* = 0.3. Compared with the previous service-life estimations obtained using *m* = 0.4, the reduction of the aging factor to *m* = 0.3 resulted in substantially lower predicted service lives for all mixtures and exposure conditions.

**Table 10:** Estimated Service Life (years) to Chloride-Induced Corrosion Initiation for Reinforced Concrete Structures, Calculated Using the Time-Dependent Form of Fick’s Second Law^a^

		Estimate of service life (years)					
Surface chloride (*C*_s_)	Environmental exposure classes (EC) adopted to determine the minimum concrete cover thickness	M1	M2	M3	M4	M5	M6
Splash/tidal zone (3.25%)	EC III–slab (35 mm)	4.6	7.0	7.4	14.2	13.7	21.6
	EC III–beams, columns, and elements in contact with soil (40 mm)	6.8	10.2	10.8	20.8	20.1	31.6
	EC IV–slab (45 mm)	9.5	14.3	15.2	29.2	28.1	44.3
	EC IV–beams, columns, and elements in contact with soil (50 mm)	12.8	19.3	20.5	39.4	38.0	59.8
Marine atmosphere near coastline (1.5%)	EC III–slab (35 mm)	11.8	17.8	18.9	36.3	35.0	55.1
	EC III–beams, columns, and elements in contact with soil (40 mm)	17.3	26.0	27.6	53.2	51.2	80.7
	EC IV–slab (45 mm)	24.2	36.4	38.7	74.5	71.7	>100
	EC IV–beams, columns, and elements in contact with soil (50 mm)	32.7	49.2	52.3	∼100	96.9	>100
Distant coastal atmosphere (0.85%)	EC III–slab (35 mm)	40.5	60.9	64.7	>100	>100	>100
	EC III–beams, columns, and elements in contact with soil (40 mm)	59.3	89.2	94.8	>100	>100	>100
	EC IV–slab (45 mm)	83.1	>100	>100	>100	>100	>100
	EC IV–beams, columns, and elements in contact with soil (50 mm)	>100	>100	>100	>100	>100	>100

a Results consider an aging factor *m* = 0.3, critical chloride threshold *C*_crit_ = 0.4%, and non-steady-state migration coefficients (*D*_ns_) at 28 days corresponding to concrete mixes M1 to M6. Service lives are presented for different surface chloride contents (average) and exposure classes.

This behavior is physically consistent with the interpretation of the aging factor in the chloride transport modeling. The parameter *m* represents the rate at which the chloride diffusivity decreases over time due to continued cement hydration, pore structure refinement, and progressive matrix densification. Higher aging factors indicate a stronger reduction in diffusivity with time, which slows chloride ingress and, consequently, increases the estimated time required for chlorides to reach the reinforcement level.

When a lower aging factor is adopted, the reduction in diffusivity over time becomes less pronounced, meaning that concrete maintains higher chloride transport kinetics throughout its service life. As a result, chloride ions penetrate more rapidly through the cover depth, reducing the predicted corrosion-initiation time. For example, for mixture M1 under splash/tidal exposure and EC III slab conditions, the predicted service life decreased from 9.2 years for *m* = 0.4 to 4.6 years for *m* = 0.3, corresponding to a reduction of approximately 50%. Similarly, for mixture M6 under EC IV beam conditions exposed to a marine atmosphere near the coastline, the predicted service life decreased from approximately 541 years to 153 years when the aging factor was reduced from 0.4 to 0.3.

The influence of the aging factor becomes increasingly significant for mixtures presenting lower chloride transport coefficients and larger concrete cover thicknesses since long-term diffusivity reduction strongly affects chloride ingress kinetics over extended exposure periods. Consequently, the aging factor constitutes one of the most sensitive parameters in diffusion-based service-life prediction models, and its selection can substantially influence the estimated durability performance of reinforced concrete structures exposed to chloride-rich environments.

Additionally, Table 11 presents the estimated minimum concrete cover thicknesses required to achieve a target service life of 50 years against chloride-induced corrosion initiation, calculated using the time-dependent form of Fick’s second law, considering aging factors *m* = 0.4 and *m* = 0.3. The results demonstrate the strong influence of both chloride transport resistance and diffusivity aging behavior on durability-based cover design. In general, mixtures presenting lower chloride migration coefficients required substantially smaller concrete covers to achieve the same target service life. Furthermore, the reduction of the aging factor from *m* = 0.4 to *m* = 0.3 significantly increased the required cover thicknesses for all investigated exposure conditions, reflecting the less pronounced reduction in chloride diffusivity over time associated with lower aging factors.

**Table 11:** Estimated Minimum Concrete Cover Thickness Required to Achieve a 50-Year Service Life against Chloride-Induced Corrosion Initiation, Calculated Using the Time-Dependent Form of Fick’s Second Law, Considering Aging Factors *m* = 0.4 and *m* = 0.3, a Critical Chloride Threshold (*C*_crit_) = 0.4%, and Non-Steady-State Chloride Migration Coefficients (*D*_ns_) Measured at 28 Days for Mixtures M1–M6^a^

Surface chloride concentration (*C*_s_)	Mixture	Required cover for 50 years (*m* = 0.4)	Required cover for 50 years (*m* = 0.3)
Splash/tidal zone (3.25%)	M1	53.2 mm	80.5 mm
	M2	46.1 mm	69.8 mm
	M3	45.1 mm	68.3 mm
	M4	35.9 mm	54.4 mm
	M5	36.3 mm	55.0 mm
	M6	31.0 mm	47.0 mm
Marine atmosphere near coastline (1.5%)	M1	38.3 mm	58.0 mm
	M2	33.2 mm	50.3 mm
	M3	32.5 mm	49.2 mm
	M4	25.8 mm	39.1 mm
	M5	26.2 mm	39.7 mm
	M6	22.4 mm	33.8 mm
Distant coastal atmosphere (0.85%)	M1	24.9 mm	37.7 mm
	M2	21.6 mm	32.7 mm
	M3	21.1 mm	32.0 mm
	M4	16.8 mm	25.4 mm
	M5	17.0 mm	25.8 mm
	M6	14.5 mm	22.0 mm

a Results are presented for different surface chloride concentrations representative of splash/tidal exposure, marine atmosphere near the coastline, and distant coastal atmospheric conditions.

When compared to the minimum reinforcement cover thicknesses prescribed by ABNT NBR 6118:2026, important differences can be observed. For reinforced concrete structures exposed to marine environments, the Brazilian standard typically recommends nominal cover thicknesses ranging from approximately 35 mm for EC III slab elements to 50 mm for EC IV beams, columns, and structural elements in contact with the soil. Under splash/tidal exposure conditions, several mixtures–particularly M1, M2, and M3–required cover thicknesses substantially greater than the normative minimum values to achieve the target service life of 50 years, especially when *m* = 0.3 was considered. In some cases, the required cover exceeded 70–80 mm, indicating that the minimum prescriptive values established by the standard may not always guarantee the intended durability performance for concretes presenting relatively high chloride transport coefficients.

Conversely, mixtures M4–M6 frequently achieved the target service life with cover thicknesses comparable to or even lower than those prescribed by ABNT NBR 6118:2026, particularly under marine atmospheric and distant coastal exposure conditions. For example, under distant coastal atmospheric exposure and considering *m* = 0.4, the required cover thicknesses for M4–M6 ranged from approximately 15 to 17 mm, substantially below the normative minimum values. This behavior demonstrates that concrete presenting refined pore structures and lower chloride diffusivity may potentially provide adequate long-term durability even with reduced cover thicknesses.

Under splash/tidal exposure conditions, which represented the most aggressive chloride environment investigated, the required cover thicknesses reached values as high as approximately 80 mm for mixture M1 when *m* = 0.3 was considered, while mixture M6 required approximately 47 mm under the same conditions. Conversely, for distant coastal atmospheric exposure, the required coverage decreased considerably, reaching values close to or below conventional durability prescriptions for several mixtures. These results reinforce the strong dependence of durability performance on both the environmental chloride severity and the long-term evolution of concrete transport properties.

Therefore, although the cover thicknesses discussed in Section 4.1 appeared to be more than sufficient when considering only the experimentally measured chloride penetration depths obtained from the accelerated migration tests, the service-life estimations based on the *D*_ns_ values produced required cover thicknesses much closer to those prescribed by ABNT NBR 6118:2026. This observation highlights the importance of combining short-term chloride penetration measurements with time-dependent transport modeling to obtain more realistic long-term durability assessments. While chloride penetration depths evaluated during accelerated testing represent only the instantaneous advancement of chlorides under specific laboratory conditions, the service-life approach incorporating *D*_ns_ values accounts for the long-term kinetics of chloride transport throughout decades of exposure.

It is worth highlighting the importance of using concretes containing supplementary cementitious materials (SCMs) since these materials may significantly improve the resistance to chloride ingress by refining the pore structure and reducing ionic transport capacity over time. In addition to lowering the chloride migration coefficients, SCM-based systems frequently exhibit higher diffusivity aging behavior due to continued pozzolanic and latent hydraulic reactions, which contribute to progressive matrix densification during the service life. Consequently, the incorporation of SCMs may reduce the required reinforcement cover thickness necessary to achieve a target durability performance, potentially enabling more durable and optimized reinforced concrete structures to be exposed to chloride-rich environments.

Lastly, a limitation of the present study is that the direct characterization of the pore structure was not performed. The interpretation of matrix densification and changes in transport pathways is therefore based on indirect indicators, including void index, water absorption, UPV, chloride penetration depth, and *D*_ns_, supported by established relationships reported in the literature.^5,9,29^ Although these parameters consistently exhibited trends compatible with changes in concrete compactness and chloride transport resistance, they cannot independently quantify the pore-size distribution, pore connectivity, or tortuosity. Future investigations should therefore complement the present approach with direct microstructural characterization, such as mercury intrusion porosimetry, microcomputed tomography, or other appropriate pore structure techniques.

## 5 Final Remarks

This study evaluated the chloride transport resistance of reinforced concrete mixtures with different water-to-cement ratios and assessed the relationship between experimentally measured chloride penetration and the minimum reinforcement cover thicknesses prescribed by ABNT NBR 6118:2026 for environmental exposure classes III and IV. Accelerated chloride migration testing was combined with compressive strength, water absorption, void index, ultrasonic pulse velocity, and Fick-based service-life modeling.

Under the accelerated chloride migration conditions adopted in this study, all investigated mixtures presented chloride penetration depths lower than the minimum concrete cover thicknesses prescribed by the ABNT NBR 6118:2026 for EC III and EC IV. Even mixtures with water-to-cement ratios exceeding the maximum values specified for the investigated exposure classes remained below the prescribed cover thicknesses during the accelerated test period. These results indicate that under the specific accelerated conditions investigated, the measured chloride penetration fronts did not reach the normative cover depths. However, this observation should not be interpreted as direct evidence that the prescribed cover thicknesses guarantee the intended long-term service life or as a definitive demonstration of the conservatism of the Brazilian prescriptive approach.

The reduction in water-to-cement ratio was associated with a substantial improvement in chloride transport resistance. Chloride penetration depth decreased from approximately 25 to 13 mm, while the non-steady-state chloride migration coefficient (*D*_ns_) decreased from 12.08 × 10^–12^ to 4.11 × 10^–12^ m^2^/s. These trends were accompanied by increases in compressive strength and UPV and reductions in void index. Because direct pore-structure characterization was not performed, these results are interpreted as indirect evidence of changes in concrete compactness and chloride transport resistance rather than direct experimental confirmation of pore refinement, pore connectivity, or tortuosity.

Strong correlations were observed between the chloride penetration depth and *D*_ns_, compressive strength, void index, and UPV. These relationships indicate that conventional mechanical, physical, and nondestructive parameters can provide useful indirect information regarding chloride transport resistance within the range of mixtures investigated. Nevertheless, these parameters should not be regarded as direct measurements of chloride transport mechanisms or pore structures.

The service-life calculations based on the time-dependent form of Fick’s second law demonstrated the strong sensitivity of the estimated corrosion-initiation time to chloride transport resistance, concrete cover thickness, surface chloride concentration, and the aging factor. The sensitivity analysis using aging factors of 0.4 and 0.3 resulted in substantially different estimated initiation times, demonstrating the influence of assumptions concerning the long-term evolution of chloride diffusivity. Several combinations of mixture and exposure condition produced estimated initiation times below 50 years, whereas substantially higher values were obtained under less aggressive assumed chloride exposure conditions.

These service-life results should be interpreted cautiously because the apparent diffusion coefficient was derived from accelerated migration coefficients obtained at 28 days using a simplified empirical conversion, while other parameters, including the aging factor, surface chloride concentration, and critical chloride threshold, were based on literature values. In addition, the Fick-based model does not explicitly account for cracking, moisture fluctuations, wetting–drying cycles, temperature variations, chloride binding, carbonation, or other field-related factors. Therefore, the calculated values represent comparative estimates of chloride-induced corrosion-initiation time under the adopted assumptions rather than definitive predictions of actual structural service life.

Overall, the results demonstrate that the experimentally measured chloride penetration under accelerated migration conditions was lower than the prescribed concrete cover thicknesses for all investigated mixtures, while the subsequent modeling showed that long-term corrosion-initiation estimates can vary substantially depending on concrete transport properties and environmental assumptions. The findings therefore support the use of accelerated migration testing as a comparative tool for evaluating chloride transport resistance while highlighting the need for long-term exposure data and experimentally determined transport parameters to improve the reliability of service-life assessments.

Future studies should complement the present approach with direct characterization of the concrete pore structure and microstructure using techniques such as mercury intrusion porosimetry, microcomputed tomography, nitrogen adsorption, and microscopic analysis. Measurements of chloride transport coefficients at different curing ages would also allow the time dependence of chloride transport to be experimentally determined and provide a more robust basis for defining the aging factor used in service-life modeling. In addition, long-term natural or seminatural marine exposure tests could help assess the applicability of accelerated migration results to field conditions. Further service-life studies should consider additional factors affecting chloride ingress, including cracking, moisture variations, wetting–drying cycles, temperature, chloride binding, and spatial variability of surface chloride concentration. These complementary investigations would help reduce the uncertainties associated with long-term extrapolation and improve the reliability of chloride-induced corrosion-initiation assessments.
